# The Native Microbial Community of Gastropod-Associated *Phasmarhabditis* Species Across Central and Southern California

**DOI:** 10.3389/fmicb.2022.903136

**Published:** 2022-07-14

**Authors:** Jacob Schurkman, Rui Liu, Salma Alavi, Irma Tandingan De Ley, Ansel Hsiao, Adler R. Dillman

**Affiliations:** ^1^Department of Nematology, University of California Riverside, Riverside, CA, United States; ^2^Department of Microbiology and Plant Pathology, University of California Riverside, Riverside, CA, United States

**Keywords:** *Phasmarhabditis californica*, *Phasmarhabditis hermaphrodita*, *Phasmarhabditis papillosa*, microbiome, gastropods

## Abstract

Nematodes in the genus *Phasmarhabditis* can infect and kill slugs and snails, which are important agricultural pests. This useful trait has been commercialized by the corporation BASF after they mass produced a product labeled Nemaslug®. The product contains *Phasmarhabditis hermaphrodita*, which has been cultured with *Moraxella osloensis*, a bacterial strain that was originally thought to be responsible for causing mortality in slugs and snails. The exact mechanism leading to death in a *Phasmarhabditis* infected host is unknown but may involve contributions from nematode-associated bacteria. The naturally occurring microbial community of *Phasmarhabditis* is unexplored; the previous *Phasmarhabditis* microbial community studies have focused on laboratory grown or commercially reared nematodes, and in order to obtain a deeper understanding of the parasite and its host interactions, it is crucial to characterize the natural microbial communities associated with this organism in the wild. We sampled *Phasmarhabditis californica*, *Phasmarhabditis hermaphrodita*, and *Phasmarhabditis papillosa* directly from their habitats in Central and Southern California nurseries and garden centers and identified their native microbial community *via* 16S amplicon sequencing. We found that the *Phasmarhabditis* microbial community was influenced by species, location, and possibly gastropod host from which the nematode was collected. The predominant bacteria of the *Phasmarhabditis* isolates collected included *Shewanella*, *Clostridium perfringens*, Aeromonadaceae, Pseudomonadaceae, and *Acinetobacter*. *Phasmarhabditis papillosa* isolates exhibited an enrichment with species belonging to Acinetobacter or Pseudomonadaceae. However, further research must be performed to determine if this is due to the location of isolate collection or a species specific microbial community pattern. More work on the natural microbial community of *Phasmarhabditis* is needed to determine the role of bacteria in nematode virulence.

## Introduction

Nematodes are one of the most ecologically diverse groups of organisms on Earth. They exist on every continent, surviving in all climates where decomposition occurs (Ingham, 1994; Bongers and Bongers, 1998; De Ley, 2006; Schafer, 2016). Some exist as free-living organisms like *Caenorhabditis elegans*, and many have evolved to form a variety of parasitic relationships like *Ascaris lumbricoides*, or the entomopathogenic nematode (EPN) *Steinernema feltiae* that has been utilized for biological control against pestiferous insects (Kaya, 1993; Riddle et al., 1997; Marilyn, 2008). EPNs have evolved specific mutualistic relationships with bacterial species in their gut that helps to kill various insects (Grewal and Georgis, 1997). Recent metagenomic analyses have indicated that the commensal microbial community of EPNs, the gut microbial community, is more complex than originally thought, leading to the possibility of a native EPN pathobiome that assists with insect killing (Ogier et al., 2020).

One nematode that has been successfully used as a biological control agent is *Phasmarhabditis hermaphrodita* (Wilson et al., 1993b). All members of the genus are gastropod-specific facultative parasites. *Phasmarhabditis hermaphrodita* was discovered in Europe and has been commercialized for biological control in Europe under the name Nemaslug®. *Phasmarhabditis* nematodes are effective at killing pestiferous gastropods in laboratory and agricultural settings such as nurseries and a variety of crops (Wilson et al., 1994a,b; Rae et al., 2007b; McDonnell et al., 2020; Tandingan De Ley et al., 2020; Schurkman et al., 2022a) but are safe to tested non-gastropod organisms (Grewal and Grewal, 2003; Iglesias et al., 2003; Rae et al., 2007a; Nardo et al., 2010). However, due to the discovery of *Phasmarhabditis* in Europe, its use has not yet been permitted in the United States since invasive species are not permitted for biological control use in the country.

It was originally thought that *Phasmarhabditis* nematodes kill their hosts in a manner similar to EPNs, which employ mutualistic and pathogenic microbes to assist with insect killing (Wilson et al., 1993b; Tan and Grewal, 2001a). This hypothesis came about upon the discovery that *P. hermaphrodita* cultured with *Moraxella osloensis* was highly pathogenic to the grey field slug *Deroceras reticulatum*, more so than when it was cultured on other bacteria (Wilson et al., 1995a; Tan and Grewal, 2001b). The selection of bacteria to test came from isolates identified in *P. hermaphrodita* infective juveniles (IJs), dead *D. reticulatum*, and xenic foam chip cultures (Wilson et al., 1993a, 1995a). The species identified and tested included *Aeromonas hydrophila*, *Aeromonas* sp., *Flavobacterium breve*, *Flavobacterium odoratum*, *M. osloensis*, *Providencia rettgeri*, *Pseudomonas fluorescens* (isolate no. 140), *P. fluorescens* (isolate no. 141), and *Serratia proteamaculans*. When these bacteria were injected into *D. reticulatum*, *A. hydrophila*, and *P. fluorescens* (isolate no. 140) caused the most mortality. However, *P. fluorescens* (isolate no. 140) was able to facilitate better growth when culturing *P. hermaphrodita*, which may be indicative of this bacterial species serving as an optimum food source. However, nematodes which were grown on *M. osloensis* exhibited the highest pathogenicity while also allowing for good nematode growth. Another experiment showed that axenic *P. hermaphrodita* did not cause mortality in *D. reticulatum* while those reared on *M. osloensis* did (Tan and Grewal, 2001b). These experiments led to the assumption that *P. hermaphrodita* likely has a natural association with *M. osloensis*, similar to EPNs’ association with pathogenic bacteria (An et al., 2008; Wilson and Rae, 2015). After these experiments, it was generally accepted among the *Phasmarhabditis* community that these nematodes caused death in their gastropod hosts strictly through the utilization of the apparent commonly associated bacteria *M. osloensis*. However, the natural association of other bacteria with *Phasmarhabditis* in the wild and how this association contributes to nematode host virulence remained largely unexplored.

In 2010, it was shown that *P. hermaphrodita* associates with many bacterial species that do not affect its virulence (Rae et al., 2010), in contrast to the existing understanding of *Phasmarhabditis* virulence (Wilson et al., 1995a). Rae et al. (2010) suggested that *P. hermaphrodita* does not associate with specific bacteria due to the observation of inconsistent and varied bacterial assemblages with the nematode (Rae et al., 2010). However, the study was unable to identify key microbial species that regularly occur within *Phasmarhabditis*. This is because a limitation to PCR-DGGE is that specific bacterial species cannot be identified. Therefore, no conclusions were made on the presence of *M. osloensis* since that was not the goal of the study. The study only aimed to show that *Phasmarhabditis* associated with multiple bacterial assemblages. In another study, bacteria were analyzed from laboratory grown *Phasmarhabditis*, by allowing nematodes to crawl on LB agar plates and identifying some of the bacterial colonies that subsequently arose (Howe et al., 2020). Eight genera of bacteria were identified that were hypothesized to have come from the laboratory grown *Phasmarhabditis*. *Pseudomonas* was the only genus found in this most recent study that was also found in 1995 (Wilson et al., 1995a; Howe et al., 2020). *Moraxella osloensis*, which was expected to be identified, was not found. These mirror findings related to the native and naturally occurring microbial community of the model organism *C. elegans* (Dirksen et al., 2016), though prolonged *in vitro* growth in the laboratory raises the possibility of association with microbes not commonly found with *Phasmarhabditis* in the wild.

Describing the natural and infected microbial community of the host could help to distinguish whether microbes present within *Phasmarhabditis* originated from the host or from another source. Very little microbial community research has been done on *D. reticulatum*, the slug often used in *Phasmarhabditis* studies (Walker et al., 1999). However, gut microbial community metagenomic analyses have been performed on other slug species like *Ambigolimax valentianus* which identified a core microbial community of *Citrobacter*, *Delftia*, *Erwinia*, *Arthrobacter*, *Stenotrophomonas*, *Pseudomonas*, *Rhodococcus*, and *Bacillus*. *Arion ater*’s microbial community was also found to be influenced by the substrate they were on, while the soil microbial community itself could also be influenced by the introduction of the slug (Jackson, 2020; Jackson et al., 2021). A gut metagenomic analyses of the slug *A. ater* has also been performed. The most abundant bacterial genera in the gut of *A. ater* included *Enterobacter*, *Citrobacter*, *Pseudomonas*, and *Escherichia* (Joynson et al., 2017).

All microbial community studies that have taken place involving *Phasmarhabditis* have used laboratory cultured nematodes, and the microbial community changes upon introduction to a laboratory environment, especially when the nematodes are grown on monoxenic cultures (Dirksen et al., 2016). Recently, three species of *Phasmarhabditis* were discovered during gastropod surveys of California nurseries and garden centers (Tandingan De Ley et al., 2014, 2016). Between 2018 and 2021 additional surveys for *Phasmarhabditis* nematodes were performed to determine the distribution of these species (Schurkman et al., 2022b). Newly-isolated nematodes collected during these most recent surveys were used to identify the natural microbial communities of *Phasmarhabditis* isolates across the Central and Southern California regions. Similarities or differences between *Phasmarhabditis* isolates could help to further the understanding of the potential role that the microbial community plays between *Phasmarhabditis* nematodes and their hosts. In this study, we aimed to determine if the *Phasmarhabditis* microbial community is species specific, and if it is influenced by gastropod hosts, geographic locality, and collection methods.

## Materials and Methods

### *Phasmarhabditis* Survey Collection

Fourteen plant nurseries from Central California and five nurseries from Southern California were surveyed for gastropods infected with *Phasmarhabditis* as described in Schurkman et al. (2022b). In short, 1 person hour was spent searching for gastropods. Then, gastropods were sorted into containers by species and taken back to the laboratory at UC Riverside in coolers. Gastropods were decapitated and placed on 1% plain agar to create seed cultures (1 L: 10 g agar, 900 ml H_2_O) and their bodies were observed for the presence of nematodes under a dissecting microscope. Upon finding a nematode(s) which phenotypically resembled a member of the *Phasmarhabditis* genus (i.e., the significant presence of phasmids), up to five individuals were placed on Nematode Growth Medium [NGM; 1 L: 3 g NaCl, 20 g Agar, 2.5 g Peptone, 975 ml deionized H_2_O, and 10 ml Uracil (2 g/L) were added to a liter of deionized water, autoclaved, and let cool, to which were added 25 ml filtered KPO_4_, 1 ml filtered MgSO_4_, 1 ml CaCl_2_, and 1 ml Cholesterol (5 mg/ml)] to create axenic cultures. All nematodes on axenic culture plates were considered as identical species of the same strain with further verification after single nematode DNA sequencing. The axenic culture plates were stored at 17°C. Individual nematodes suspected to be *Phasmarhabditis* were picked from axenic culture plates and were prepared for PCR and DNA sequencing of the D2-D3 domains of the rDNA LSU, as described in Tandingan De Ley et al. (2014). Contigs were assembled and compared by BLAST with published sequences on Genbank using CodonCode Aligner (CodonCode Corp., 58 Beech Street, Dedham, MA, United States) to identify nematode species. A percent identity match near 99% was required in order to indicate a species identification using BLAST or CodonCode Aligner.

### *Phasmarhabditis* Treatment and Storage

All nematodes suspected to be a member of the *Phasmarhabditis* genus *via* microscopy were prepared for microbial community analysis. This preparation was done for each nematode picked from gastropod cadavers and before axenic culture plates were created. To prepare, nematodes in seed cultures which phenotypically matched those that were used for axenic culture were subjected to a rinse. When possible, at least one nematode from each seed culture was washed in sterile M9 buffer (1 L: 3 g KH_2_PO_4_, 6 g Na_2_HPO_4_, 5 g NaCl, 1 ml 1 M MgSO_4_, and H_2_O to 1 L) thrice and then placed inside of 10 μl of sterile H_2_O in a 200 μl PCR tube, which was stored at −20°C for future use. These nematodes were labeled as washed nematodes. The washes were performed to rinse excess material from the cuticle of the nematode. When possible, at least one nematode was also not subjected to any treatment at all, and the nematode was immediately picked and placed inside of a 200 μl sterile microtube with 10 μl of sterile H_2_O and stored at −20°C. These nematodes were labeled as unwashed nematodes. Lastly, 10 μl of dead and partially decomposed gastropod tissue was pipetted into 10 μl of sterile H_2_O in a 200 μl PCR and was stored at −20°C. Comparisons to unwashed nematodes and decomposed slug tissue were done to observe whether the washes significantly altered the microbial community of *Phasmarhabditis*. Upon finding a *Phasmarhabditis* nematode *via* 28S sequencing, we used the corresponding nematode(s) previously frozen at −20°C for microbial community analysis.

### DNA Extraction

Genomic DNA was extracted from all washed and unwashed nematodes, as well as from the decomposed dead gastropod tissue. The DNA extraction protocol included thawing samples on ice and breaking the individual nematodes into pieces within their PCR tube using a sterile 10 μl pipette tip. After breaking the nematodes, the total volume of all samples was brought up to 100 μl with sterile PCR grade water. An equal volume of phenol chloroform was then added to each sample. The contents of the small PCR tubes were then transferred to a 1.25-ml Eppendorf tube and were mixed *via* pipettor. The tubes were shaken by hand for 30 s and were then centrifuged at 12,000 rpm for 10 min at 4°C. After centrifugation, the aqueous phase of the solutions was removed and placed in a new 1.25-ml Eppendorf tube. The wash with phenol chloroform was repeated once more, and then 400 μl of isopropanol stored at −20°C was added to the solution. A 1:10 ratio of 3 M sodium acetate was then added to the solution and was mixed *via* pipetting up and down. The tubes were then shaken by hand for 30 s and to each tube, 1 μl of glycogen stored at −20°C was added before storing at −20°C for 24 h. The samples were then centrifuged at 13,000 rpm for 30 min at 4°C to form a pellet. All liquid was then removed from the tubes with a pipettor, being careful not to disturb the pellet at the base of the tube. Using a pipette, the pellet was carefully washed with 500 μl of ethanol which was immediately drawn from each tube. The tubes were then stored at 37°C in an incubator until no visible liquid was present. The pellet was then resuspended in 50 μl of sterile PCR grade water and the DNA was quantified using a Qubit 3 Fluorometer (Invitrogen by Thermo Fisher Scientific and life technologies, Waltham, MA, United States).

### 16S rRNA Gene Library Preparation and Sequencing

The bacterial 16S rDNA V4 region 515F-806R was amplified according to the earth microbial community project, 16S Illumina protocol (Thompson et al., 2017). Based on the concentrations of each single nematode DNA sample, 1–8 μl of the extracted DNA template, 10 μl Platinum Hot Start PCR Master Mix (ThermoFisher), and 0.5 μl of forward and reverse primers (10 μM) were added into the 25 μl PCR reaction system, with the barcode in the reverse primer. Thermocycler conditions were 4°C for 3 min, followed by 30 cycles (94°C for 45 s, 50°C for 60 s, 72°C for 90 s), and 72°C for 10 min. PCR products were pooled together and submitted to an Illumina MiSeq platform with 2 × 150 bp read lengths.

### Data Analysis

Raw reads were processed using the open-source software QIIME2 (Bolyen et al., 2019) version 2021.2. Samples that had >1,000 reads were retained and denoised using dada2 with default settings. Taxonomic classification was performed using classify-sklearn command against the Greengenes 13_8 99% OTUs from 515F/806R region of sequences (MD5: 9e82e8969303b3a86ac941ceafeeac86) set trimmed to 250 bp of the V4 hypervariable region (McDonald et al., 2012; Bokulich et al., 2018; Robeson et al., 2020). An amplicon sequence variant (ASV) was defined as a group of sequences with a similarity of 100%. Alpha and beta diversity analyses were calculated in QIIME2 with rarefied sample depth of 1,000, and results were plotted in GraphPad Prism 9. The heatmap was generated using pheatmap package in R program, samples and species clustered using hclust.

The statistical calculations used in QIIME2 were: Kruskal-Wallis test for alpha diversity comparisons, and permANOVA for beta diversity. Mann–Whitney U tests were performed in GraphPad Prism 9 for taxa comparisons.

## Results

### DNA Amplification and Total Reads

Of the total 146 samples from three different nematode species from various gastropod hosts collected during surveys between 2019 and 2020, 26 were amplified and sequenced successfully (Supplementary Table 1). In total 475,226 raw reads were obtained from 26 samples. A total of 397,685 high-quality reads were clustered into 337 ASVs at 100% similarity level. Twenty-two samples with read depth > 1,000 remained for subsequent analyses (Supplementary Table 1).

### Alpha Diversity Analyses

**Figure 1:**
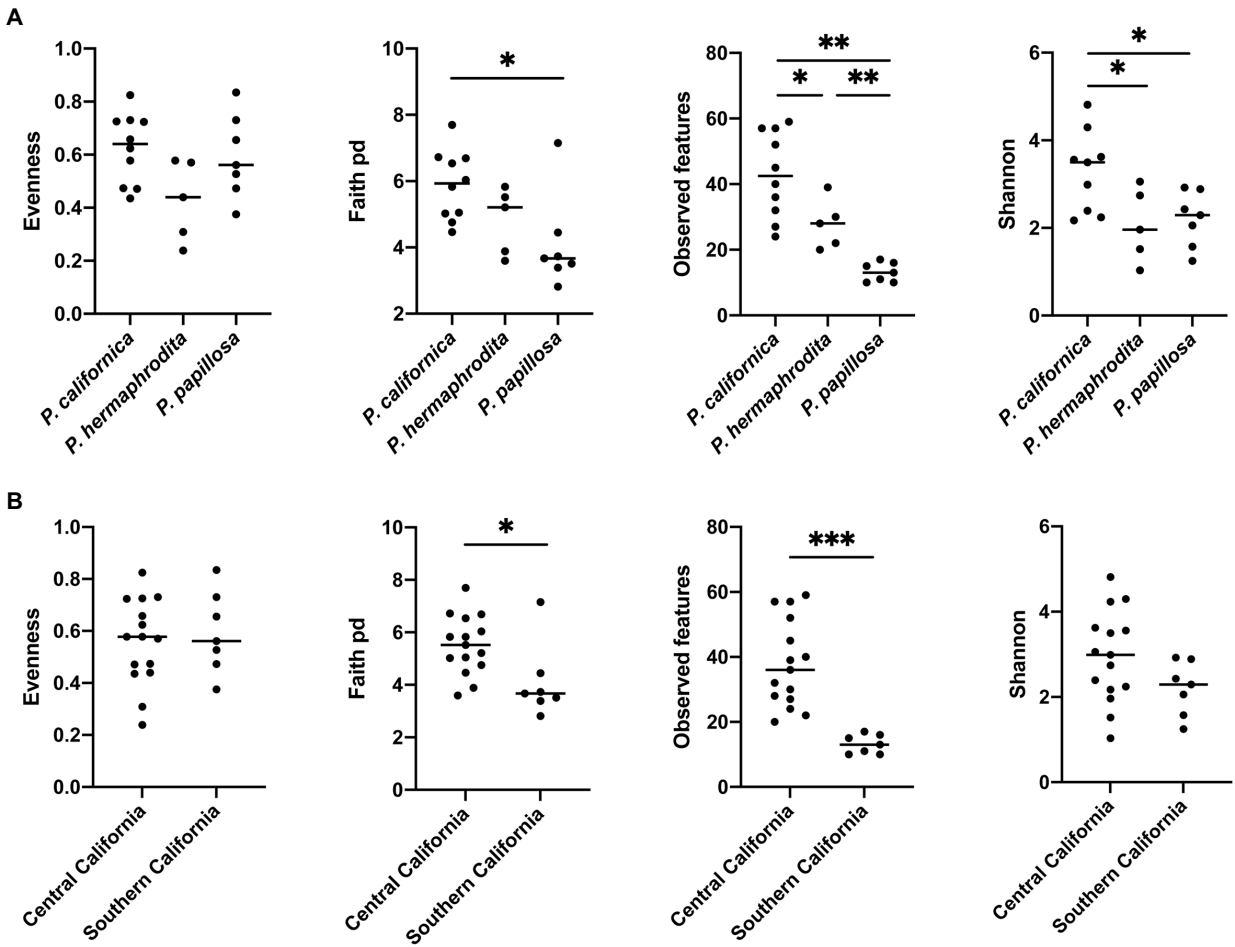
The comparison of microbial community alpha diversity of nematode-associated microbial communities. **(A)** *Phasmarhabditis* species and **(B)** location affect the richness of the microbial composition in nematode. Kruskal-Wallis test, *p < 0.05; **p < 0.01; and ***p < 0.001.

**Figure 2:**
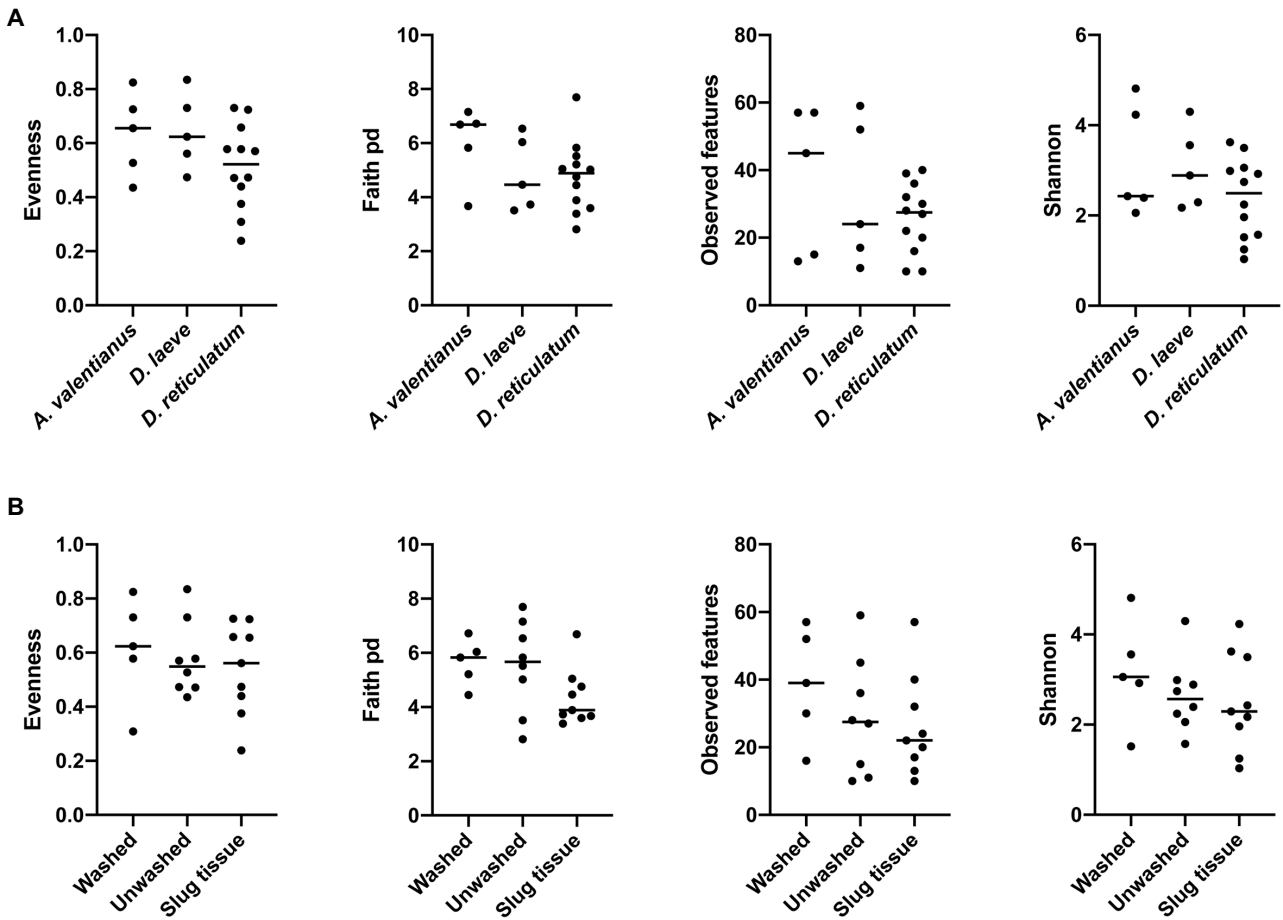
Comparison of microbial community alpha diversity across different host species or sample collection strategy. **(A)** Gastropod host and **(B)** collection strategy are not associated with differences in the diversity of the *Phasmarhabditis* microbial community.

Alpha diversity analysis showed that nematode species may be an important factor associated with the diversity of nematode microbial community’s. According to observed features, Shannon index, and Faith’s phylogenetic diversity (Faith pd), *P. californica* microbial community’s exhibited higher richness than those of *P. hermaphrodita* and *P. papillosa* (Figure 1A). Meanwhile, Central California samples had significantly higher observed features and Faith pd. index than Southern California. However, this may reflect the fact that all nematodes collected from Southern California were *P. papillosa*, which had the lowest diversity of observed features (Figure 1). The host of the nematode was not associated with differences in microbial richness or evenness (Figure 2A). No alpha diversity differences were noted across nematodes that were washed in M9 thrice, unwashed, or collected from decomposed gastropod tissue (Figure 2B).

### Microbial Community Structure Comparisons

**Figure 3:**
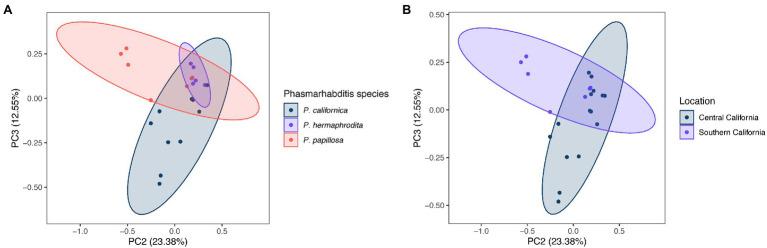
Principal coordinate analysis (PCoA) plots of nematode microbial community’s based on Bray Curtis distance. PCoA plots showing **(A)** *Phasmarhabditis* species and **(B)** location. Percent variance explained is shown in parentheses for each axis. Ellipses show 95% CI.

**Figure 4:**
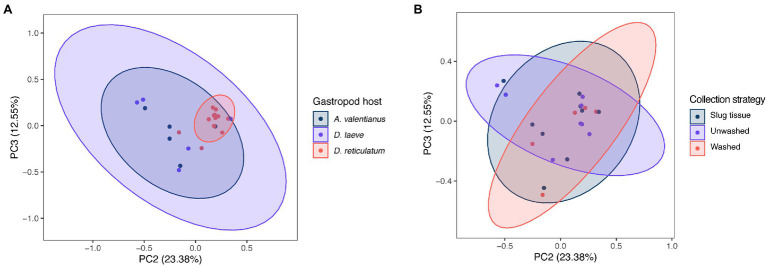
PCoA plots of nematode microbial community of different gastropod hosts, based on Bray Curtis distance. PCoA plots with samples clustered by **(A)** gastropod host and **(B)** collection strategy; % variance explained shown in parentheses for each axis. Ellipses show 95% CI.

The difference of overall microbial community structure showed similar trends with alpha diversity analyses. The PCoA plots based on Bray–Curtis distance between sample microbial community’s revealed a significant separation of bacterial composition depending on *Phasmarhabditis* species, especially between *P. californica* and *P. papillosa* (*q* value = 0.015), while the *P. hermaphrodita* microbial community overlapped with the other two species (Figure 3A), suggesting that *P. hermaphrodita*’s microbial community shared some of the same bacterial features with both *P. californica* and *P. papillosa*; these differences were statistically significant when tested using permANOVA (Table 1). Geographical location also contributed to differences in the nematode microbial community (Figure 3B), while treatment by washing with M9 did not (Figure 4B). The gastropod host also showed a slight overall association (*p* = 0.017) with the nematode microbial community (Figure 4A; Table 1), but no pairwise comparison of the hosts showed significantly separation. From these data, we conclude that nematode species and location may play an important role in shaping the native *Phasmarhabditis* microbial community and perhaps the gastropod microbial community.

**Table 1:** permANOVA analysis reveals the microbial differences between gastropod hosts, locations, *Phasmarhabditis* species, or washed/unwashed/slug tissue.

	Overall value of *p*	Group 1	Group 2	Pseudo-F	*p*	*q*
Gastropod host	***p* = 0.017**	*Ambigolimax valentianus*	*Deroceras laeve*	0.834	0.578	0.578
		*Ambigolimax valentianus*	*Deroceras reticulatum*	2.569	**0.02**	0.06
		*Deroceras laeve*	*Deroceras reticulatum*	2.109	0.056	0.084
Location	***p* = 0.007**	Central California	Southern California	3.028	**0.007**	**0.007**
*Phasmarhabditis* species	***p* = 0.003**	*Phasmarhabditis californica*	*Phasmarhabditis hermaphrodita*	1.989	0.093	0.093
		*Phasmarhabditis californica*	*Phasmarhabditis papillosa*	3.148	**0.005**	**0.015**
		*Phasmarhabditis hermaphrodita*	*Phasmarhabditis papillosa*	2.303	0.056	0.084
Collection strategy	*p* = 0.61	Slug tissue	Unwashed	0.861	0.49	0.735
		Slug tissue	Washed	1.249	0.255	0.735
		Unwashed	Washed	0.473	0.877	0.877

Bolded values indicate statistical significance.

### Taxonomic Composition of the Nematode Microbial Community

**Figure 5:**
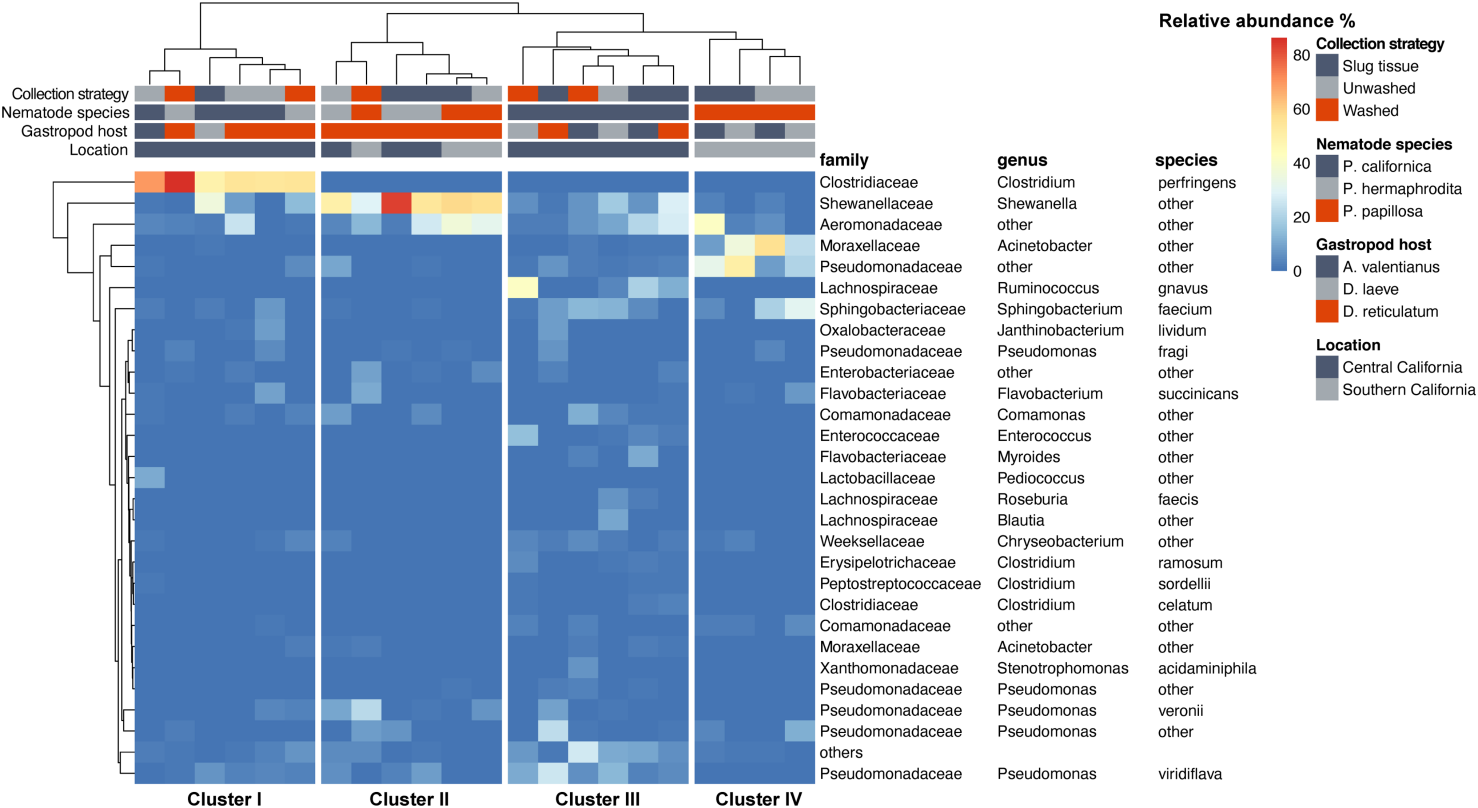
Heatmap of the nematode microbial community at species level. Species with relative abundance >5% across all samples are displayed.

**Figure 6:**
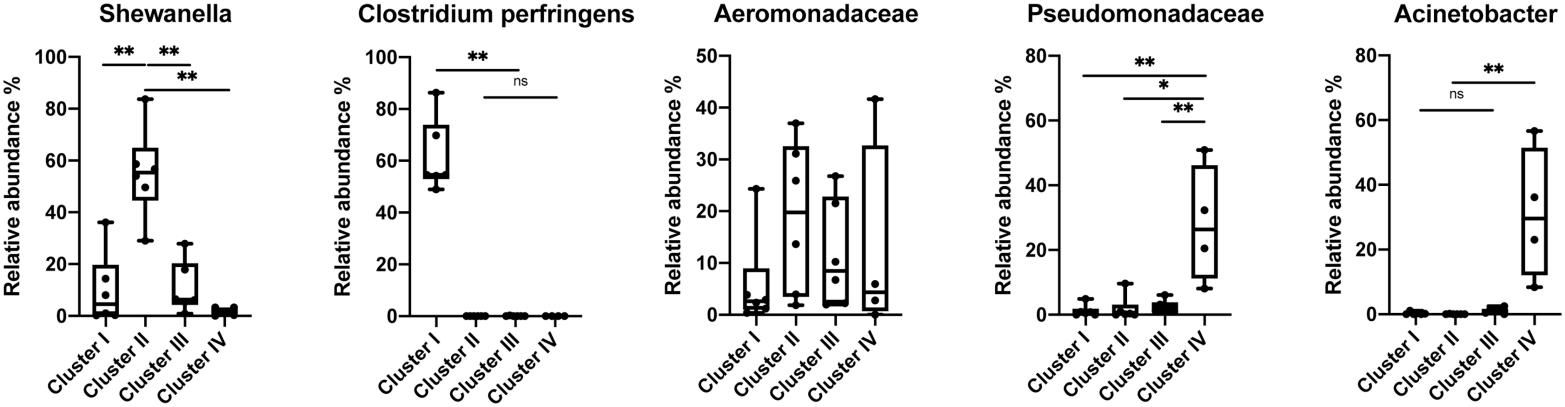
Relative abundance of the most abundant species in the *Phasmarhabditis* species. Mann–Whitney U-test, *p < 0.05; **p < 0.01. Boxplots show inter-quartile range, whiskers minimum to maximum.

The species-level bacterial community composition in the nematodes was analyzed using the unsupervised hierarchical cluster analysis. For this analysis, all the samples were divided into four groups (Figure 5). Cluster IV consisted of *P. papillosa* samples from Southern California and exhibited enrichment with species belonging to genus Acinetobacter or family Pseudomonadaceae. Cluster III consisted of *P. californica* from Central California. Cluster II consisted of a mixture of *P. papillosa* from Southern California and *P. hermaphrodita* from Central California, which were all collected from the same gastropod host, *D. reticulatum*; in these microbial communities, members of genus *Shewanella* and family Aeromonadaceae were the dominant microbial members. No members of the genus *Moraxella* were identified. Cluster I microbial samples were dominated by a high proportion of *Clostridium perfringens*, though these samples were collected from multiple nematodes and gastropod hosts from Central California. Among the most abundant species, *Shewanella* sp. was significantly increased in cluster II compared to the other clusters; samples in cluster I had 48%–86% of *C. perfringens*, which was not shown in any other clusters; species from family Pseudomonadaceae and genus *Acinetobacter* were significantly enriched in cluster IV; while family Aeromonadaceae was evenly distributed in all clusters (Figure 6).

## Discussion

This study is the first analysis of the native microbial community of *Phasmarhabditis*. It assessed the microbial community of *Phasmarhabditis* in multiple nursery and garden center habitats and aimed to help identify core microbial communities of *Phasmarhabditis* utilizing 16S metabarcoding analysis. The previous *Phasmarhabditis* microbial community work had been done using nematodes which had been kept in culture, leaving the possibility of the nematode’s microbial community being altered by laboratory conditions and frequent transfers (Wilson et al., 1993a, 1995b; Rae et al., 2010; Dirksen et al., 2016;Howe et al., 2020; Sheehy et al., 2022). Two other studies have used next generation sequencing techniques, however the studies did not use newly-recovered *Phasmarhabditis*, but instead utilized laboratory sub-cultured and maintained *Phasmarhabditis* (Howe et al., 2020; Sheehy et al., 2022). The most recent study by Sheehy et al. (2022) showed that *Phasmarhabditis* does not exclusively associate with a single bacterial species which it relies on for virulence. However, the study did not utilize native *Phasmarhabditis* and only utilized *Phasmarhabditis* nematodes which have been frequently cultured and transferred in laboratories (Sheehy et al., 2022). While some of the recent studies may suggest that *Phasmarhabditis* does not maintain a set core microbial community, understanding what microbes are naturally and commonly associated with *Phasmarhabditis* may help to solidify whether *Phasmarhabditis* utilizes any core microbial species which contributes to gastropod killing, similar to microbial contributors to EPN virulence. The findings may also reveal crucial bacterial species needed for *Phasmarhabditis* food consumption, survival, or host–parasite interactions. Our results suggest that the location, species, and possibly the gastropod host may affect the microbial diversity within the tested *Phasmarhabditis*.

Our findings are not entirely congruent with the previous *Phasmarhabditis* microbial community work. Similar to previous studies, we identified *Acinetobacter* and *Pseudomonas* spp. occurring on *Phasmarhabditis*, however, no previous studies identified predominant bacteria like *Shewanella*, Aeromonadaceae, or *C. perfringens* which were identified in this study (Wilson et al., 1995a; Rae et al., 2010; Howe et al., 2020; Sheehy et al., 2022). Pseudomonaceae and *Acinetobacter* species were enriched in some clusters of *Phasmarhabditis* nematodes, specifically cluster IV which consisted of *P. papillosa* (Figure 6). *Acinetobacter* and Pseudomonaceae bacteria are commonly found in the soil and have been discovered in multiple gastropod species (Ducklow et al., 1981; Wilson et al., 1993a; Ekperigin, 2007; Villena et al., 2010; Joynson et al., 2017; Howe et al., 2020; Jackson, 2020). It was previously found that unhealthy *Biomphalaria glabrata* snails had a core microbial community predominantly made up of *Acinetobacter* and *Moraxella* spp.; however, healthy snails had a microbial community predominantly made up of *Pseudomonas* spp. (Ducklow et al., 1981). It is possible that *Phasmarhabditis* and gastropods thrive with *Pseudomonas* spp., and the presence of other species like *Acinetobacter* or *Moraxella* spp. (the bacterial species used to mass produce Nemaslug®) in *Phasmarhabditis* cause increased pathogenicity. However, this hypothesis is disputed from a finding that showed that rearing *Phasmarhabditis* on *Acinetobacter* had no effect on its virulence (Nermut et al., 2014). The interesting pattern in which only *P. papillosa* (discovered only in Southern California) have both increased Pseudomonaceae and *Acinetobacter* needs more study. This pattern may be due to the bacterial diversity and population and potential dominance at the collection site, or a species-specific relationship with *P. papillosa*. However, another possibility is that *Phasmarhabditis* uses some of the predominant bacterial species as a major food source, and others as contributors toward virulence, or perhaps some bacteria are used as both food and a driver for pathogenesis. Since *Acinetobacter* and Pseudomonaceae are frequently found in soils and are not commonly known as highly virulent bacteria, it is possible that these predominant bacteria are used as a food source rather than enhance or contribute to pathogenicity. This hypothesis is further supported by the observation that *Phasmarhabditis* grew exceptionally well on agar cultured with *P. fluorescens* (isolate 141) or *P. fluorescens* PSG strain compared to other bacterial species. The *Pseudomonas* bacteria was still not selected for use in the commercial production of Nemaslug® possibly because it was not associated with the highest mortality rates, suggesting its role as a food source for the nematodes rather than a source of virulence (Wilson et al., 1995a,b).

*Shewanella* has been discovered in multiple gastropod species where it causes increased pathogenicity, however all studies which identified this were performed in aquatic environments (Cai et al., 2006; Wang et al., 2008). The finding of an association of *Shewanella* with *Phasmarhabditis* has not previously been reported. The bacteria were not detected in any *P. californica* isolates (Figure 5). This may have been due to a limited sample size throughout the study, or due to a random association of bacteria with *Phasmarhabditis*, as hypothesized in 2010 (Rae et al., 2010). However, *P. californica* had the largest representation throughout this study with a total of 10 utilized isolates (Supplementary Table 1). The occurrence of this predominant species may be indicative of it being used as a source of virulence toward the gastropod host; however further research is needed to assess this possibility.

Multiple gastropod species have been found associated with *Clostridium* bacteria (Charrier et al., 2006; Li, 2012). However, like *Shewanella*, the species *C. perfringens* had not previously been found in *Phasmarhabditis* or other nematodes. *C. perfringens* is most well known as a causative agent of food poisoning in mammals (Labbe, 1991). The species is frequently searched for and reported in foods for the sake of public health. There are over 1 million cases of poisoning from *C. perfringens* each year (Grass et al., 2013). A previous study demonstrated that *C. perfringens* enterotoxin could cause intestinal illness of mammals, and potentially fish and frogs (Robertson et al., 2010). However, it is not known how this bacterium affects gastropods and nematodes. *Phasmarhabditis* nematodes may serve as vectors for *C. perfringens*, using the bacteria as a weapon to kill their gastropod host. However, this seems less likely since it has been found that some gastropods can naturally harbor and vector the bacteria. It was previously thought that *C. perfringens* was only capable of reproducing in mammals and other endothermic organisms, and therefore only these organisms could vector the pathogenic bacteria. More recently it was found that ectotherms such as gastropods, frogs, and fish can also vector the bacteria and therefore these organisms should be monitored as sources of contamination (Frick et al., 2018). Our finding of these bacteria furthers the claim that ectotherms, specifically gastropod-associated nematodes, can act as vectors. However, it was only discovered in cluster I which consisted of *P. californica* and *P. hermaphrodita*. It is possible that only *P. californica*, and *P. hermaphrodita* use *C. perfringens* as a source of virulence. However, further study is needed.

The most predominant bacterial family found throughout all *Phasmarhabditis* species was Aeromonadaceae. This family was found in similar abundance across all species and served as the only predominant commonality within the genus. The family has not previously been found within *Phasmarhabditis* and is not common in many nematodes, but it has been discovered within multiple gastropod species (Villena et al., 2010; Li et al., 2019). It is possible that *Phasmarhabditis* largely assumes the microbial diversity of its gastropod host. However, this hypothesis needs further experimentation. Since Aeromonadaceae is not commonly known to be highly pathogenic to a variety of organisms and it was the most predominant species across all *Phasmarhabditis* nematodes, it can be hypothesized that *Phasmarhabditis* species utilize the bacteria as a major food source rather than a source of virulence. *Phasmarhabditis hermaphrodita* and other members of the genus are known to be bacterivorous (Tan and Grewal, 2001b). However, their food preferences at collection sites are unknown. *Phasmarhabditis hermaphrodita* has been found to grow well when reared on monoxenic cultures of *P. fluorescens*, but this does not prove its preferred bacterial food source in a native setting (Wilson et al., 1995b). Further experimentation may draw out explanations for the clustering of Aeromonadaceae observed in clusters III and IV within *P. californica* and *P. papillosa* (Figure 5).

Future work to assess the microbial diversity of *Phasmarhabditis* needs to utilize next generation sequencing technology and nematodes which have not been maintained in culture for a long period of time. Further microbial community work with the species from this study (*P. californica*, *P. hermaphrodita*, and *P. papillosa*) should be done in order to obtain more isolates for statistical power in identifying the microbial community of the species. Further study would also lead to the possibility of work with less discrepancies in read counts as we observed. Study of other *Phasmarhabditis* species microbial community should also be assessed in order to determine other species-specific microbial patterns. It is likely that the maintenance of nematodes in culture on specific media can influence the microbiota (Dirksen et al., 2016). It was recently found that *C. elegans* native microbial community differs from the previously described microbial community, and its microbial community has some consistencies across time at the genus level but can be influenced by various substrates and present bacteria. Interestingly, one of the consistent genera in *C. elegans* is *Pseudomonas*, which was one of the predominant bacteria we identified which may serve as a major food source for *Phasmarhabditis* (Dirksen et al., 2016; Johnke et al., 2020). To understand the natural relationships and mechanisms between *Phasmarhabditis* and their hosts, native isolates must be utilized. Next generation sequencing technologies allow for rapid sequencing and identification of these isolates and their microbial communities upon collection, allowing for easy assessment of the native microbial flora and their potential interactions.

## Data Availability Statement

The datasets presented in this study can be found in online repositories. The names of the repository/repositories and accessionnumber(s) can be found in the article/Supplementary Material. The 16S sequence data has been deposited in NCBI under BioProject PRJNA816167.

## Author Contributions

JS, IL, AH, and AD: conceptualization; JS, RL, SA, and AH: methodology and software; JS, RL, AH, and AD: validation and original draft preparation; JS, RL, and SA: formal analysis and investigation; IL, AH, and AD: resources and funding acquisition; JS, RL, and AH: data curation; JS, RL, SA, IL, AH, and AD: writing; JS, RL, IL, AH, and AD: writing—review and editing; JS and RL: visualization; and AH and AD: supervision and project administration. All authors have read and agreed to the published version of the manuscript.

## Funding

The work was supported by the United States Department of Agriculture Specialty Crop Multi-State Program (USDA-SCMP) in partnership with CDFA (2018–2022 grant #12509488), a generous gift (2019–2022) from CANERS Foundation through the California Association of Nurseries and Garden Centers (CANGC), Plant California Alliance (PCA), and National Institutes of Health GM124724.

## Conflict of Interest

IL is a co-inventor on a patent entitled Mollusk-killing Biopesticide (WO2017059342A1).

The remaining authors declare that the research was conducted in the absence of any commercial or financial relationships that could be construed as a potential conflict of interest.

## Publisher’s Note

All claims expressed in this article are solely those of the authors and do not necessarily represent those of their affiliated organizations, or those of the publisher, the editors and the reviewers. Any product that may be evaluated in this article, or claim that may be made by its manufacturer, is not guaranteed or endorsed by the publisher.
